# 

**Published:** 1856-01

**Authors:** 


					﻿JANUARY, 1856.
^Flllfll ^FflistFF
®f . WIST.
gks z ft
JAMES TAYLOR, M. D., D. D. S., Editor.
Published Q_<n.z-,? .. ^3 per.annum.

CINCINNATI:
WRIGHTSON & CO., PRINTERS,
167 Walnut Street.
				

